# INDOOR AIR QUALITY: SHS Plus Ozone Poses One Fine Particle Problem

**DOI:** 10.1289/ehp.118-a472

**Published:** 2010-11

**Authors:** Adrian Burton

**Affiliations:** **Adrian Burton** is a biologist living in Spain who also writes regularly for *The Lancet Oncology*, *The Lancet Neurology*, and *Frontiers in Ecology and the Environment*

Smokers might want to think about creating a no-ozone zone as researchers report that the gas can react with chemicals in secondhand smoke (SHS) to produce ultrafine particles less than 100 nm in diameter.^1^ “Given the very large surface area and the very high alveolar deposition fraction of such particles, their potential to cause health problems cannot be ignored,” says first author Mohamad Sleiman, a chemist with the Environmental Energy Technologies Division of Lawrence Berkeley National Laboratory (LBNL).

SHS contains at least 250 known toxicants,^2^ but until this study little was known about what is formed when SHS molecules react with ozone. Highly reactive ozone is a pervasive outdoor pollutant. It also is purposely produced indoors by certain air-purifying devices, ostensibly to remove airborne toxicants and odors like those from cigarette smoking (the actual effectiveness of these devices is debatable, however^3^). “We found that when the molecules in SHS react with ozone they can make ultrafine particles containing high-molecular-weight nitrogenated species,” Sleiman explains.

The researchers generated SHS by letting 10 cigarettes smolder for 15 minutes in an environmental chamber of about the volume of an 8- × 10-foot room. Then they pumped the contaminated air into 100-liter Tedlar® bags and added ozone-containing air to reach an initial ozone concentration of 110 ppb. The authors noted that the mass concentrations of both SHS and ozone used were representative of indoor environments where tobacco smoking occurred regularly and ozone air purifiers were in use.^1^ The particulate matter in the resulting mixture was sized using a scanning mobility particle sizer, and its composition was examined using a time-of-flight mass spectrometer.

“What we found was surprising: large amounts of ultrafine particles, roughly eight times higher than those present in freshly emitted tobacco smoke,” says coauthor Hugo Destaillats, also of LBNL. “Mass spectrometry showed these to be at least partly composed of high-molecular-weight nitrogenated oligomers that were not present in the original SHS.” Indeed, he says, initial SHS compounds with a mass-to-charge ratio (a kind of molecular fingerprint) of less than 370 were much reduced in the postreaction sample while many new compounds with mass-to-charge ratios of around 400 to 500 had formed.^1^

Similar experiments performed with pure nicotine also produced ultrafine particles that contained some but not all of the same new compounds, showing that many of the oligomers had formed through reactions involving other components of SHS. However, the products of nicotine ozonolysis included many molecules with asthma hazard indices much higher than that of nicotine itself.^1^ With a 4–9% total aerosol yield (the absolute aerosol mass) for the ozone–nicotine reactions alone, constant smoking could soon build up ultrafine particle concentrations in indoor air.

In recent years ultrafine particles have received increasingly bad press. Small enough to be inhaled deep into the lungs, where they can cross into the blood stream, they have been linked to a range of respiratory and cardiovascular problems through oxidative stress.^4^ Indeed, ultrafine particles can enter cells themselves and even enter mitochondria, where oxidative stress is thought to damage the cristae.^5^

“The ‘thirdhand’ smoke products made by these reactions of SHS compounds with ozone would not just be inhaled,” comments Jonathan Winickoff, an associate professor of pediatrics at Harvard Medical School. “After depositing on objects they could be absorbed through the skin or even ingested. Young children who explore the world by putting things in their mouths would be at greatest risk for this oral exposure route. When inhaled, these types of ultrafine particles place children at higher risk of asthma attacks.” The full health implications of children’s oral exposure are not yet well understood.^6^

A further problem would be the potential of these ultrafine particles to persist as residues on surfaces—perhaps for weeks—from which they could reenter the air over time, Sleiman says. “The reactants in smoke could also stick on surfaces, continuing to spawn ultrafine particles as they come into contact with ozone,” adds Gary Cohen, a senior research scientist at the Karolinska Institute. “Smokers might therefore continue to poison the indoor environment, especially for infants and children, long after they have finished their cigarette.”

## Figures and Tables

**Figure:**
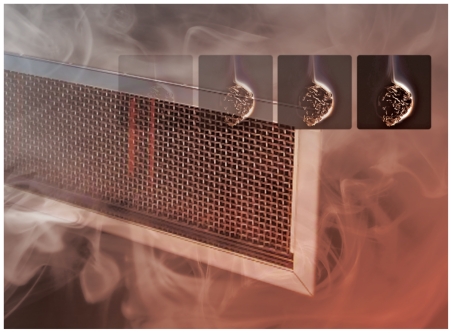
Ozone air purifiers for commercial and residential use are sold to remove cigarette smoke toxics from indoor air and thus improve its quality. Ironically, the machines could be having exactly the opposite effect.

## References

[1] Sleiman M (2010). Atmos Environ.

[2] Secondhand Smoke: Questions and Answers [website].

[3] Ozone Generators That Are Sold as Air Cleaners [website].

[4] Madl AK, Pinkerton KE (2009). Crit Rev Toxicol.

[5] Li N (2003). Environ Health Perspect.

[6] Sleiman M (2010). Proc Natl Acad Sci USA.

